# Impedance Analysis to Evaluate Nutritional Status in Physiological and Pathological Conditions

**DOI:** 10.3390/nu15102264

**Published:** 2023-05-10

**Authors:** Angela Catapano, Giovanna Trinchese, Fabiano Cimmino, Lidia Petrella, Margherita D’Angelo, Girolamo Di Maio, Marianna Crispino, Gina Cavaliere, Marcellino Monda, Maria Pina Mollica

**Affiliations:** 1 Department of Biology, University of Naples Federico II, 80126 Naples, Italy; angela.catapano@unina.it (A.C.); giovanna.trinchese@unina.it (G.T.); fabiano.cimmino@unina.it (F.C.); margheritadangelo.dangelo@studenti.unicampania.it (M.D.); marianna.crispino@unina.it (M.C.);; 2 Centro Servizi Metrologici e Tecnologici Avanzati (CeSMA), Complesso Universitario di Monte Sant’Angelo, 80126 Naples, Italy; 3 Department of Experimental Medicine, University of Campania “Luigi Vanvitelli”, 80138 Naples, Italy; girolamo.dimaio@unicampania.it (G.D.M.);; 4 Department of Pharmaceutical Sciences, University of Perugia, 06123 Perugia, Italy; 5 Task Force on Microbiome Studies, University of Naples Federico II, 80138 Naples, Italy

**Keywords:** bioelectrical impedance analysis, body cell mass, body composition, intracellular water, extracellular water, fat mass, fat-free mass

## Abstract

A thorough knowledge of body composition assessment techniques is the cornerstone for initiating a customized nutritional program. The second step is to consider the potential of their application in different physiological and pathological conditions and their effectiveness in the management of a monitoring pathway during dietary interventions. To date, bioimpedance analysis is the most effective and reliable method for assessing body composition due to its advantages in terms of speed of execution, non-invasiveness and low cost. Therefore, this review article aims to analyze the main concepts and application areas of bioimpedance measurement techniques, in particular vector frequency-based analysis (BIVA) systems, in order to assess their validity in both physiological and pathological conditions.

## 1. Introduction

Bioimpedance analysis has been used in clinical applications since the 1980s, and nowadays it is one of the most widely used tools for investigating body composition, universally accepted as an essential element in the assessment of an individual’s health status [1]. It is currently the most effective and reliable methodology for estimating body composition and fluid status in the body. Its use has increased significantly over the past decade for its undoubted advantages, such as rapidity of execution, non-invasiveness, and low cost. The acronym BIA stands for Bioimpedance or Bioelectrical Impedance Analysis, where bioimpedance and bioelectrical impedance are used interchangeably [1]. BIVA, on the other hand, stands for Bioelectrical Impedance Vector Analysis [1].

Various methods are used to interpret the data and there is a wide range of uses for bioimpedance in body composition estimation. More than 1600 articles have been published on this methodology since the 1990s. Although the literature has recognized a wide variety of specific applications, clinicians have been slow to adopt a practical use of BIA technology. Interestingly, from 2000 to 2006, the number of articles cited in Medline containing the keywords ‘bioimpedance’ or ‘impedance tomography’ increased by 56 percent.

The electrical properties of tissues have been known for a long time (suffice it to say that Emperor Tiberius used electrical discharges made from fish to relieve pain) and described since 1871 [2]. Therefore, while the therapeutic uses of electric current have a long and well-established history, its use as a diagnostic tool has encountered technical difficulties related to measurement accuracy, electrical model complexity in human tissue, and correct interpretation of results. The most striking example is electrocardiography, which took about 60 years, starting from its first demonstration in 1887, to be accepted as a valid diagnostic tool. The first insight into the diagnostic use of impedance dates back to 1888, by Vigouroux, director of Salpetrière’s electrotherapy. The history of the use of electric current to explore and find information about tissue composition and function draws on a number of influential contributions that, to date, form the basis of bioimpedance analysis. Thomasset [3] explored the use of bioimpedance measurement in estimating total body water (TBW) using needle electrodes [4]. Nyboer [5] applied quadruple surface electrode readings for bioimpedance measurements to estimate human lean body mass (FFM). Hoffer [6] introduced the association between total body impedance and total body water content with reference to tritium dilution techniques. In 1969 he and his collaborators proved through a series of experiments that TBW and bioelectrical impedance are highly correlated, suggesting that impedance measurements can be used to determine TBW. Two experimental groups were tested and compared: the first consisting of 20 healthy subjects and the second of 34 patients with different levels of hydration. The results showed a very high correlation (r = 0.92) between the impedance index (stature^2^/R) and TBW. The impedance index equation derived is the one used to date in BIA analysis [6]. The impedance meter, the instrument used for bioimpedance analysis, was first commercialized in 1979 by Eng. Rudolph J. Liedtke, founder of RJL System, which even today, more than 40 years later, remains a leading company in the engineering and development of BIA applications. The instrument involved electrodes, attached on the back of the right hand and at the end of the right foot, which measured impedance by passing a stream of 50-kHz current through the right half of the body. The method had immediate success mainly because of its easy application. Until then, in fact, the only ways to quantify body composition were by caliper and underwater weighing [7].

The first application of BIA occurred in the military, in 1981 on Mount McKinley, Alaska. Dr. William Mills MD, an admiral in the U.S. Navy, using Hoffer’s 1969 study [6], which stated that hand-to-foot BIA measurements could predict TBW, investigated the hydration status of soldiers in particularly harsh climates, and at high altitudes. Four BIA instruments were ordered by the U.S. Navy from RJL Systems, specially designed and built to handle the cold at the top of Mount McKinley, about 6000 m above sea level. At the same time, in the same environment and temperatures, the blood and urine parameters of the soldiers were monitored [8]. The electrodes were placed as we are doing today. A few years later, Hank Lukaski of the University of North Dakota was one of the first to publish a paper on BIA and body composition [9]. Currently, bioimpedance testing can be applied to many fields:

- Primary care medicine: monitoring of the subject’s nutritional status, especially in conditions of obesity or overweight; detection of pathologies related to fluid variation in the body; evaluation of hydro-electrolyte changes aimed at early diagnosis of heart failure; dosage of diuretics [10].
- Sports medicine: checking body hydration and monitoring changes in FFM and fat mass (FM); detecting changes as a result of intense training or inadequate diet [11].
- Obstetrics and gynecology: monitoring weight changes and water retention in pregnancy and menopause [12].
- Geriatrics: monitoring changes in weight and dehydration with aging, with an assessment of dietary and hydration needs.
- Dietetics: monitoring changes in body compartments during specific dietary programs with an assessment of hydration status and cell mass.
- Artificial nutrition: assessment of the individual’s initial nutritional status for setting the most appropriate nutritional program, also for the intensive care and resuscitation room.
- Nephrology and dialysis: identifying the patient’s ideal dry weight to tailor the hemodialysis session; monitoring body fluids to investigate the need for dialysis; and assessing pre- and post-dialysis water status [13].
- Oncology and HIV: identification of patients with reduced cell mass or altered water balance from therapies; phase angle as a prognostic index of survival [14].

## 2. Body Composition

All body composition analysis methods aim to return valid results under both physiological and pathological conditions. When properly used, BIA is able to capture changes in tissue hydration [1]. BIA is basically the study of the electrical properties of biological material and its change over time, but its applications have been focused on human body composition. BIA can be used in clinical, educational, or research settings. Body composition assessment offers health professionals and patients a powerful and accurate window into disease and health [15]. BIA is based on the principle that biological tissues behave as conductors, semiconductors, or insulators (dielectrics). The intra- and extracellular electrolyte solutions of lean tissues are good conductors, while bone and fat are dielectric substances, so electric current does not pass through them [16,17,18,19].

In the healthy population, TBW is around 60% of weight; however, this value undergoes changes depending on several factors, such as age, amount of adipose tissue, and hormonal changes. TBW can be divided into intracellular water (ICW), which is contained within cell membranes, accounting for about two-thirds of the total, and extracellular water (ECW), which can be further divided into interstitial and intravascular fluids [20]. BIA analysis is based on the passage of an alternating current flow through biological materials, which act as conductors. The opposition to the passage of current, known as impedance (Z), can be measured and decomposed into two electrical components: resistance (R) and capacitive reactance (X_c_). R is determined by tissues as conducting materials of the current through intra- and extracellular electrolyte solutions. This value is closely associated with fluid content, the main conductors of the human body; therefore, low R is associated with large amounts of body fluids while high R relates to lower fluid levels. When the % of body water increases, the conductivity of the body increases. Since body water is mainly contained within FFM, the conductivity of the body is proportional to the amount of the FFM itself [9].

X_c_, known as capacitive, R is the force opposing the passage of electric current at the level of a structure with capacitance, that is, a capacitor. In the human body, the cell membrane, a lipid (insulating) structure interposed between two fluid- and protein-rich (conducting) environments, is an excellent model of a capacitor. The cell, therefore, is the component of the body endowed with reactance to the passage of electric current, accumulating negative charges on the cytoplasmic side and positive charges on the extracellular. X_c_ is thus an indirect measure of cell membranes and is used to estimate Cell Mass (BCM). Impedance (Z) is the vector sum of R and X_c_. It is important to point out that the measurement of Body Impedance Z alone is not sufficient for the correct estimation of body composition because fluid-related bioelectrical values are accumulated with those related to cell membranes and thus to BCM [21,22].

**Figure 1:**
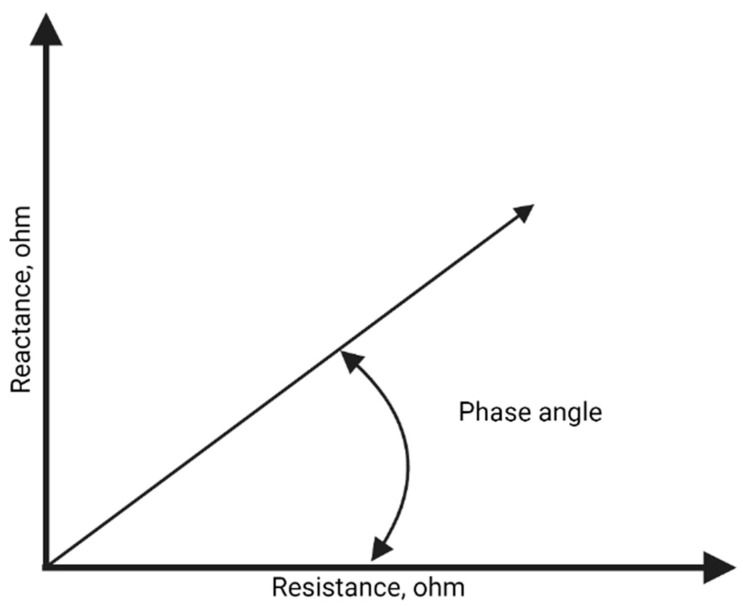
Geometric relationships between resistance, reactance, and phase angle in living organisms. Impedance is the vector composed of resistance and reactance. The phase angle describes the position of the impedance vector and depends on the values of resistance and reactance [23].

Phase angle (Figure 1) is an indicator of physical state and cellular integrity. It is derived from the ratio of the two components of the vector: Tan^−1^ (X_c_/R).

A vast body of research on thousands of subjects has shown that the relationship between Phase angle and cellular health increases almost linearly. A high Phase angle may be related to large amounts of BCM or dehydration states [24,25,26]. X_c_ is higher in subjects with good cellular mass and diminishes with age. This occurs because advancing age and the onset of disease produce changes in electrical conductivity and cell membrane permeability and/or correlate with changes in ICW and BCM. R behaves inversely by increasing with decreasing body fluids and decreasing with increasing body fluids [27]. Thus, by analyzing the resistive Rz and capacitive X_c_ vectors and their Phase angle ratio, we obtain reliable estimates of body composition as well as a detailed analysis of FFM.

The normal Phase angle value is between 5° and 9° depending on the age and sex of the subject. A high Phase angle is associated with good physical condition, while a low Phase angle is usually associated with malnourished or pathological subjects [23].

Therefore, while the Z impedance of a healthy and decompensated subject might be similar, the evaluation of bioelectric vectors (R and X_c_), through the Phase angle, instead allows us to assess the differences in physical state, hydration, nutrition, and, therefore, overall health. In fact, malnourished or decompensated subjects have a Phase angle lower than normal values [21,22,23,25,26,28].

The impedance meter is a device capable of running a flow of charges through a biological conductor, specifically the human body.

The resistance of an object is determined by a formula, which is described as length (L) and surface area (A), and by the type of material, which is described by resistivity (ρ), as shown in the following equation [29]:

(1) $$R_{\left(\Omega \right)}=\rho_{\left(\Omega \cdot m\right)}\;\frac{L_{\left(m\right)}}{A_{\left(m^{2}\right)}}$$

**Figure 2:**
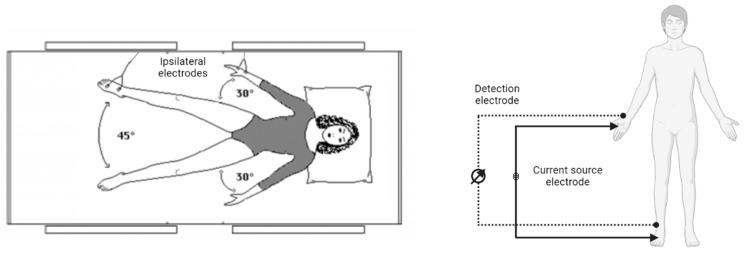
Illustration of electrode connection and wiring diagram of current flow [1,21].

The empirical relationship is between FFM (about 73% water) and height^2^/R. Due to the inhomogeneity of the intrinsic field in the body, the term height^2^/R describes an equivalent cylinder, which must match the actual geometry by an appropriate coefficient. This coefficient depends on various factors, including the anatomy of the segments under examination. Therefore, errors occur in the presence of alterations in the resistivity of the conductive material. Two pairs of electrode pads applied to the right hemisoma are used for measuring the bioimpedance vector Z, although it is possible, for special needs, to use the left. Each pair consists of an injector electrode and a sensor. This system allows quick measurements (on the order of five minutes, including the application of the electrodes), and is noninvasive, harmless, repeatable, and inexpensive, with the advantages of portability and without interference with other health electrical instruments. Specifically, a pair of electrodes is applied on the back of the hand between the two protruding bones and about 1 cm away from the joint (metacarpophalangeal joint) of the middle finger, precisely the injector electrode on the metacarpophalangeal joint of the third finger and the sensor electrode on the radio-ulnar joint. The two electrodes should be at least 5 cm apart. The other pair is applied on the ipsilateral dorsum of the foot, one on the metatarsophalangeal joint of the third toe (injector) and the other on the tibiotarsal joint (sensor). Again, it is important to maintain a distance of 5 cm between the two sensors. The subject, lying supine, must not wear metal elements or come in contact with them, and has to keep the ipsilateral hand and foot uncovered; lower limbs are spread 45° and upper limbs 30° to maintain enough distance from the trunk. The analyzer leads are connected with tweezers to the electrodes (Figure 2).

In standard BIA, an alternating current pulse of intensity absolutely harmless to tissue (between 500 and 800 μA) is applied at a frequency of 50 kHz. The values of R and X_c_ are displayed in real-time by the analyzer.

Standard BIA measures global tissue hydration as an average value of segmental and compartmental components (intra- and extracellular) with weighting factors varying between subjects and between clinical conditions. The body is assumed to be integrated as an isotropic conductor (i.e., exhibiting the same physical characteristics in all directions) with a constant cross-sectional area; the reliability of the estimates is progressively reduced when the subject moves away from a state of normal hydration, and muscle mass, and this constitutes its limitation. In fact, a constant soft tissue hydration of 73% is assumed. Therefore, the correlations between the various body compartments are constant and interdependent, in the absence of hydro-electrolyte alterations, so that the various body compartments can be quantitatively assessed. However, in subjects with alterations caused by water retention, edema, and dehydration, the standard error is always too high, and BIA use in any clinical field is inhibited.

**Figure 3:**
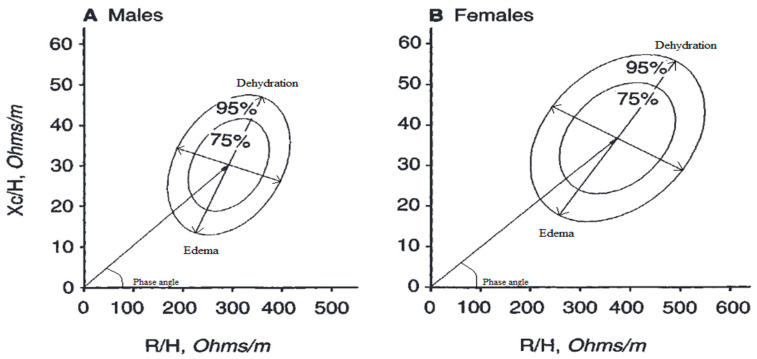
BIVA model for classifying soft tissue hydration and mass. R is the resistance, X_c_ is the reactance, and H is the height. Reprinted with permission from Piccoli et al. [26] © 1994, International Society of Nephrology.

In vector BIA, which uses direct measurement of body impedance, the two measurements R and X_c_ are considered simultaneously as components of the impedance vector Z on the RX graph (Figure 3).

A subject’s vector, normalized for stature, is compared graphically with the distribution of vectors in the healthy reference population; 95% confidence ellipses for mean vectors and 50%, 75%, and 95% tolerance ellipses for single vectors are used. Using a universal correlation coefficient, r = 0.64, a single Z-score graph was obtained with the three tolerance ellipses (50%, 75%, and 95%) centered on zero, with dimensionless axes ZI and Z(X_c_), at multiple values of the standard deviation with scale in the range +/− 5 SD. The transformation to Z score is obtained with the two equation I(R) = (R − R _mean_)/SD and Z(X_c_) − (X_c_ − X_c mean_)/SD [1,9,15].

**Figure 4:**
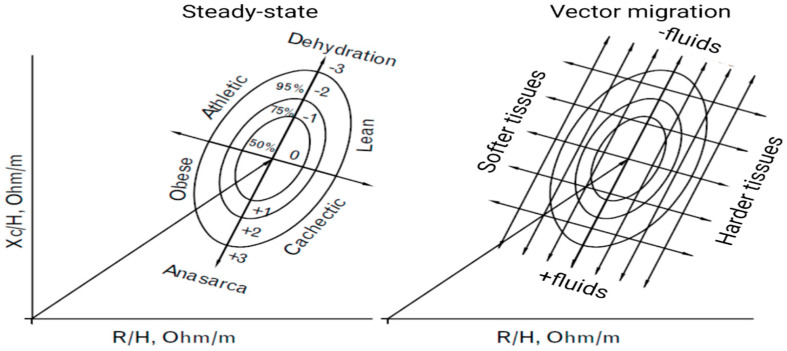
Bioelectrical impedance vector analysis and RX_c_ plot.: top left are people with good muscle mass, bottom left are obese, top right are very thin subjects and anorexics, and bottom right are cachectic [30]. Reprinted/adapted with permission from Piccoli et al. [26]. © 1994, International Society of Nephrology.

Vector BIA can capture changes in hydration through parameters independent of body weight [13] and without any mathematical regression equation of the data, thus without assuming constant hydration at the outset. Piccoli et al. [13,25,26] developed a BIVA approach influenced only by impedance measurement error and biological variability of subjects. In BIVA, R, and X_c_, standardized for height, are plotted as point vectors in the R-X_c_ plane. A single vector can then be compared with the 50%, 75%, and 95% reference tolerance ellipses calculated in the healthy population of the same sex and race (R-X_c_ plot method). The ellipse varies with age and body size [29] (Figure 4).

A single individual measurement can be compared with confidence ellipses of a healthy population, and a normal individual impedance vector is expected to fall within the 75% reference tolerance ellipse [24,26]. Repeated measurements in the same individual can be evaluated by shifts in the vector. A shortening or lengthening of the vector above the confidence ellipses signifies fluid overload (edema) or dehydration, respectively. If the vector increases or decreases its Phase angle, it can be interpreted as more or less cell mass. By combining the analysis of Phase angle with vector length, it is possible to differentiate obese (short vector, high Phase angle) from athletic individuals (long vector, high Phase angle) on the left side of the RX_c_ graph and lean (long vector, low Phase angle) from cachectic individuals (short vector, low Phase angle) in the right side of the graph. The great advantage of this method is that it allows information on changes in tissue hydration or soft tissue mass to be obtained simultaneously, independent of any regression equation or body weight [31]. The BIVA measurement could therefore be a preliminary test for the use of regression equations; individual vectors outside the 75% tolerance range of a reference ellipse may have unsatisfactory results from the BIA equations [32].

Factors influencing the impedance vector Z are mainly gender, ethnicity, and age [33].

Gender: Variations in body composition between males and females have been demonstrated in several studies. In the prediction of body composition, methods based on bioimpedance analysis, and most equations tend to include gender as a major determinant in the assessment of body compartments [21]. Studies on FFM show that males have higher FFM than females with different ranges. The average FFM for males is 8.9 kg and for females 6.2 kg; the fat mass index FMI increases with age, in females from 5.6 to 9.4 and in males from 3.7 to 7.4. Due to the different body composition between males and females, gender considerations have a strong impact on estimating body compartments, and it can be said that, for all age groups, males have less FM and more FFM than females.

Ethnicity: From studies conducted in the U.S., Caucasians have average vectors with equal R/H and reduced X_c_/H compared with those of African Americans and Mexican Americans with the same BMI. However, the impedance vector of females is found to be longer and with the same Phase angle. Body composition varies among different races and ethnic groups due to environment, nutritional factors, culture, and anthropometric measurements that include body conformation. There are also differences in limb length, body structure, and body size, and this leads to variations in body fat percentages among different ethnic groups that can cause prediction errors (3%). Therefore, validation of bioimpedance measurements among different ethnic groups is needed because of differences in body composition among some populations.

Age: Aging is a multifactorial change in the physical and biological activities of the human body that leads to differences in body composition among age groups. As the human body ages, there is a gradual increase in FM and a concomitant decrease in FFM; a significant increase in average weight is associated among the elderly population compared with adults with an increase in FM. In the Italian reference population, aged 18 to 85 years, with BMI from 17 to 31 kg/m^2^ evenly distributed by age, the correlation between R/H, X_c_/H, and age fluctuates around the value 0, both in males and females. Weak correlations tend to be positive between age and R/H, and negative between age and X_c_/H. This bipolarity of correlation between the two components of the vector with age influences the position of the vectors: older vectors on the right and the younger ones on the left, along the minor axis of the ellipses. In large case histories, this effect of age on the distribution of vectors is present in the 50–59 age group and above, at the same BMI. If a young person’s vector falls in the right hemiellipse, it means that the soft tissue mass is less than expected. The vector of an elderly person falling into the left hemiellipse indicates a greater soft tissue mass than expected by age. The bipolarity of the correlation between age and vector components causes a greater negative correlation between Phase angle and age [1,9,15].

In essence, a shorter vector indicates higher tissue fluid content; a longer vector indicates lower fluid content. Lengthening and shortening of the vector, parallel to and near the main axis, indicate changes in fluids with conserved cytotissue structure.

What is provided, therefore, is qualitative information. By inputting the data obtained into specific software where they are used in regression formulas, estimation at the quantitative level (kg, l, %) is possible for:

- TBW: its measurement is obtained from the ratio of stature (H) squared to R (impedance index, H^2^/Z or resistance index H^2^/R) to which other variables are added to increase the accuracy of the regression (multiple).
- ECW and ICW
- FFM
- FM
- BCM
- Muscle mass (MM)

**Figure 5:**
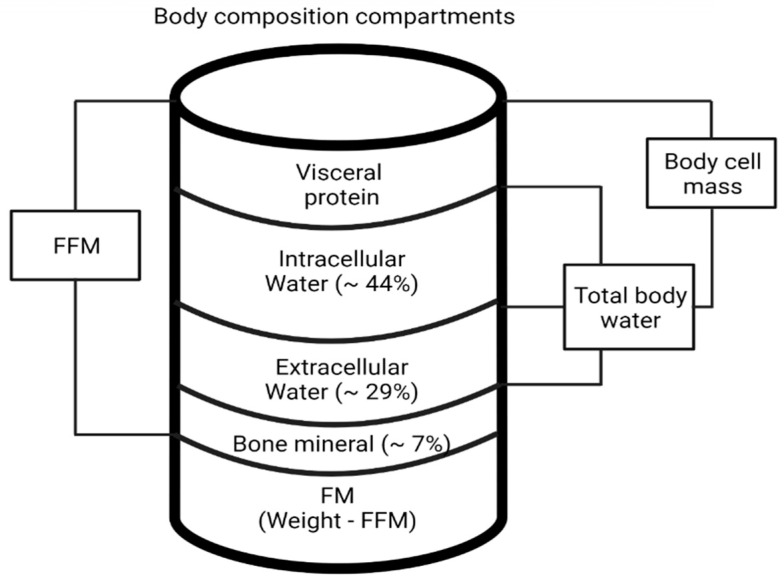
Schematic diagram of lean body mass (FFM), total body water (TBW), intracellular water (ICW), extracellular water (ECW), and body cell mass (BCM) [21].

A schematic diagram of the body composition compartments is provided in Figure 5.

Whole body BIA measures various segments and is related to several factors, such as hydration and fat fraction. Thus, the validity of simple empirical regression models is very limited. Localized BIA, on the other hand, is able to focus the analysis on defined and circumscribed body segments, minimizing interference effects. Some studies [30] have determined local abdominal fat mass by localized BIA; others [34] have ascertained, in patients with neuromuscular disease, that the Phase angle and limb resistivity decrease with disease progression and normalize with disease remission, proving the validity of the method in the therapeutic evaluation of such diseases [21].

## 3. BIA Application under Physiological Conditions

### 3.1. Sports and Muscle

Getting the most correct estimate of ECW and BCM is of great help for proper nutrition and training planning. In sports, especially competitive sports, it is important to set up personalized training plans. Only by knowing the athlete’s body composition is it possible to assess hydration status and muscle mass so as to prepare the best training regimen for each athlete.

The ratio of FM to FFM becomes crucial in highly athletically demanding disciplines performed at a competitive level. Therefore, the term “diet” in professional sports does not refer to dietary restrictions but rather to a qualitative and quantitative organization of caloric intake aimed at the preservation and increase of FFM. Fluid balance is therefore of the utmost importance as effective hydration corresponds to much faster recovery, especially after intense performance [35,36,37]. The main fields of application in sports, competitive as well as amateur, are both evaluative (fitness condition of the athlete, monitoring of muscle anabolism and supercompensation during the preparation phase, response to workload, recovery management) and preventive (onset of fatigue, dehydration, possible injuries). In particular, it is applied in:

- Programming proper training for a better choice of workloads and recovery times
- Nutrition programming
- Checking hydration status
- Intra- and extracellular fluid monitoring
- Evaluation of Phase angle as an index of general physical state
- Assessment of BCM to verify improvements and to avoid super-training states
- Assessment of basal metabolic rate and energy expenditure.

Proper assessment of body composition allows individual sports aptitudes to be defined and tailored to the individual in order to achieve the best competitive performance through targeted training techniques and methods. In fact, weight assessment alone is not a suitable method to distinguish changes in body composition. The application of impedance testing in sports is varied and ranges across all disciplines, from dance to soccer to being used in the assessment of the nutritional status of bodybuilders [11,36].

The field method BIA has shown promising reIuIin its ability to predict TBW and accurately estimate FM and FFM, but research on athletes is limited and the validity of bioimpedance to estimate FM and FFM in addition to TBW is unclear. In addition, the ability of bioimpedance to accurately track changes in body composition is uncertain [38]. Classical and specific BIVA has been shown to be very effective in assessing maturity-related differences in body composition of young athletes embarking on a competitive career [35,37]. A group of 178 players between the ages of 12 and 16 from an Italian Serie A professional soccer team was divided into three groups according to maturity status. Bioelectrical resistance and reactance were assessed on all of them. Fat mass percentage (FM%) and TBW were estimated through bioelectric values. TBW was higher (*p* < 0.01) in adolescents classified as “early” maturity status than in the other two groups, and classical BIVA confirmed these results. Thus, the study provided new classical and specific BIVA reference values of 50%, 75%, and 95% for elite male youth soccer players. TBW increases as somatic maturation progresses, while FM% remains stable. Considering the BIVA models, early maturing athletes show shorter carrier lengths and a tendency for higher Phase angles than timely and late maturing peers [35].

For the purpose of obtaining an athlete-specific profile based on body composition parameters and providing reference bioelectrical impedance values for men’s volleyball players, a group of 201 athletes enrolled in Italian volleyball divisions was studied, divided into an elite group (75 players participating in the 1st Super League division), a sub-elite group (65 athletes playing in the A2 Series) and a low-level group (61 players participating in the B Series). The athletes’ bioelectrical impedance, body weight, and height were measured in the second half of the competitive season, and BIVA was performed. The elite group presented higher FFM and TBW and lower FM than the subelite group (*p* < 0.05). In addition, the elite players were taller and heavier and had higher FFM, FM, TBW, and body cell mass than the subelite athletes (*p* < 0.05). Finally, the mean impedance vectors of the elite group differed significantly from those measured in the other two groups and obviously in the normal population. Thus, the study provided a set of reference values of body composition and bioelectrical impedance of elite men’s volleyball players, useful for the interpretation of individual bioimpedance vectors and for defining target regions for these athletes [37].

The assessment of prope” muscle function is not only of interest in the sports arena but in healthcare where it has gained considerable diagnostic and clinical importance. A commonly used method of assessing muscle function is the measurement of hand grip strength. This parameter has also been shown to be useful diagnostically as it is predictive, much more than weight loss alone, of possible postoperative complications and surgical risk. It is also a superior prognostic parameter compared with biochemical and anthropometric markers of nutritional status. However, although hand grip strength measurement is a simple method to estimate muscle function, patient cooperation in terms of complicity and ability is still required. Therefore, it is important to find alternative methods that reflect function without needing the patient’s consciousness and active cooperation. The impedance parameters R/H and X_c_/H correlate with hand grip strength, and vector migration in the RX_c_ graph is associated with increased grip strength. Of great interest is the possibility of assessing a clinically relevant parameter, such as muscle function, even without patient cooperation [36].

### 3.2. Pregnancy and Menopause

Pregnancy expects the maternal body to automatically adapt to the demands of fetal growth. It is a condition where drastic physical changes in body composition occur within a very narrow time frame. These include weight gain, adipose tissue, and TBW, or both intracellular and extracellular, and vary from woman to woman [39]. The increase in TBW during pregnancy contributes to the pregnant woman’s weight gain in the range of 50 to 70%. Specifically, the increase in TBW in pregnancy results from a number of concomitant causes (fetus, placenta, amniotic fluid), but also from the expansion of plasma volume, with a peak of change in the cardiovascular system during the second trimester. Women with pre-eclampsia have been shown to have a greater amount of TBW than healthy pregnant women [40].

The bioimpedance technique Ias proven in recent decades to provide a breakthrough in the assessment of body fluid changes that occur throughout pregnancy. The classical methods of body water assessment used until then (e.g., isotope dilution with deuterium or oxygen-18) were actually complicated in execution, invasive, and expensive. There is evidence for a strong correlation between estimates of body fluid composition using the isotope dilution technique and those obtained through BIA [39]. This evidence, combined with the simple execution, noninvasiveness, and low cost, have made BIA, and especially BIVA, the election technique for body water assessment during pregnancy. Evaluating bioelectric indices, it is possible to monitor hemodynamic adaptation and identify patients at risk for the onset of hypertensive pathology in pregnancy [41]. Changes in body composition, particularly those related to the distribution of FM in women, occur not only during pregnancy, but also during the pubertal period and, in particular, during the menopausal transition.

In menopause, the most noticeable features are a decrease in FFM and an increase in FM as a percentage of body weight. These changes are influenced by age and changes in hormone concentration. Menopause is related to the aging process associated with loss of bone mass, muscle mass, strength, and overall physical performance. Women are more prone to these changes than men. These changes in BCM have significant health implications and correlation with chronic disease, immobility, and fracture risk.

In addition to the loss of FFM, hormonal deficits occur in women, and adipose tissue changes its distribution, localizing mainly at the abdominal level (central- or android-type obesity). Clinically, abdominal-type obesity correlates with an increased risk of type II diabetes, dyslipidemia, high blood pressure, certain types of cancers, and, of course, cardiovascular disease [42,43,44,45]. Therefore, menopausal and postmenopausal weight gain appears to be significantly influenced by and correlated with physiological and behavioral changes associated with age, in addition to hormonal changes [46]. Regardless of hormonal status, visceral obesity is associated with an altered cardiovascular risk factor profile in women [47].

Weight fluctuation in menopause is related to strong changes in the hydro electrolyte framework. Hormone replacement therapy, which reduces adiposity on the trunk and also in the lower part of the body, actually changes the body sections and with them the impedance response. Thus, the impedance equation adopted before therapy may no longer be valid in post-treatment due to two factors: the changed body morphology and the changed density of FFM. Certainly, in menopause, the redistribution of adipose tissue may alter the proportions of individual body districts and result in the need for a specific predictive equation for the post-menopausal period [48].

### 3.3. Aging

Caloric-protein malnutrition increases with age among both men and women. For hospitalized patients, caloric-protein malnutrition affects 30 to 60% of individuals, reaching 85% in long-term care facilities or nursing homes. Women are found to be more prone to severe depletion. In economically advanced countries, protein-energy malnutrition (PEM) occurs almost exclusively in the elderly population. The causes of malnutrition in the elderly are numerous and, in independent living conditions, can be divided into medical causes such as chronic bronchitis, emphysema, failed dentition, difficulty in swallowing, and social causes such as loneliness, irregular meals, isolation, and economic difficulties. Instead, in the hospital or long-stay residential setting, malnutrition may be related to factors completely unrelated to the individual, including a lack of sufficient interaction between physician, dietitian, and OSS staff, as well as a lack of emphasis on nutrition education in medical schools [49]. Anthropometric measurements, i.e., surveying weight and height should be part of the clinical evaluation of all elderly individuals.

The negative consequences of malnutrition can be reduced with the early identification of at-risk situations, and through the development of appropriate nutritional support and supplementation strategies. The main ones are:

- Subjective Global Assessment (SGA)
- Sadness, Cholesterol, Albumin, Loss of weight, Eating problems, Shopping problems, or Inability to prepare a meal (SCALES)
- Geriatric Nutritional Risk Index (GNRI)
- Nutrition Risk Score (NRS)
- Nutrition Screening Initiative (NSI)
- Nutritional Risk Assessment Scale (NuRAS)
- Mini Nutritional Assessment (MNA)

Supported by the validity of the aforementioned protocols, and in particular of SALES and MNA [50], BIA, especially BIVA, has emerged as an indispensable investigative and diagnostic aid in relation to the detection of weight and changes in elderly subjects, also in order to prepare, in addition to the most correct dietary program, a targeted exercise program.

Several studies suggest that age is one of the major factors contributing to bone fracture risk. In older adults is documented a decrease in bone mineral density (BMD) [51], the parameter for determining the severity of osteoporosis, which is defined as a low bone mass associated with the appearance of microarchitectural changes in the bone [52]. In addition to aging, postmenopausal osteoporosis also results in a change in mineral bone density due to a loss of trabecular bone as a result of changes in body composition and hormone levels [53].

The gold standard method to assess BMD is dual X-ray absorptiometry (DXA). However, the DXA scanner has shown some disadvantages including high costs and limited availability, and, therefore, may result not be suitable for a large-scale study. Studies showed that BIVA outcomes including PhA, height-adjusted R (R/H), and X_c_ (X_c_/H), were related to BMD levels in the whole body, spine, and proximal femur in the elderly population. In particular, a low PhA is associated with osteoporosis. This provided a new biomarker for BMD using BIVA [51]. Accordingly, previous studies have already demonstrated the existence of a correlation between BIA and BMD [53,54].

## 4. BIA Application in Pathological Conditions

### 4.1. Overweight and Obesity

Obesity represents a serious health emergency that affects an increasing number of individuals, no longer mainly in developed countries, but also in developing countries, and in segments of the population that until a few years ago had low manifestations of this pathology, namely the elderly population. In fact, by increasing life expectancy, and thus, the number of individuals in the third age, the prevalence of obesity in this particularly fragile segment of the population has also increased, and it requires specific tools for diagnosis and targeted therapeutic approaches. According to World Health Organization data in developed countries, obesity is one of the top 10 health risk factors [55]. Obesity increases the risk of many non-communicable diseases, including cancer, cardiovascular disease, type 2 diabetes mellitus, and chronic respiratory disease. It has also been observed that people with obesity have an increased risk of complications and mortality in the case of SARS-CoV-2 infection [56]. However, obesity is a complex disease whose causes are very diverse and do not simply result from the combination of inadequate diet and physical inactivity. According to the WHO report, the latest scientific evidence presents vulnerability to inadequate body weight in early life as an influence on the future tendency to develop obesity, as well as environmental factors typical of highly digitized societies as potential causes [56].

In order to validate the use of bioelectrical impedance in the assessment of body composition in relation to obesity, a study was conducted on 87 adults with body composition varying in the range of 8.8–59.0% FM by subjecting them to bioelectrical impedance measurement and underwater weighing (density). Density-determined lean body mass (FFMd) was compared with FFM estimated from bioelectrical impedance, according to regression equations. Correlation coefficients were high at all levels of FM (0.94–0.99), except when FM was more than 42%. In this case, the impedance equations overestimated FFM compared with FFMd. This effect was greater in subjects with more than 48% FM, and a regression equation was derived to determine FFM for these subjects [57]. Conventional body composition methods may produce a biased quantification of FM and FFM in obese subjects due to the possible violation of the assumption of constant (73%) tissue hydration. Another study [58] involved 540 obese subjects with body mass index (BMI) >31 kg/m^2^, without apparent edema, compared with 726 healthy subjects with BMI <31 kg/m^2^ and 50 renal patients with apparent edema. A subgroup of 48 obese subjects was re-evaluated after weight loss (8.6 kg, 3 BMI units) after a month-long energy intake restriction (1200 kcal/d) and 32 obese uremics were evaluated before and after a dialysis session (3.2 kg fluid removed).

As a result of bioelectrical measurements, it was deduced that: impedance vectors of obese subjects could be discriminated from those of edematous patients falling within the ellipse containing the mean vectors with 95% probability; significant vector elongation occurs in obese subjects after the fluid loss of 3 kg; weight loss of about 9 kg after restrictive diet regimen is not associated with any vector shift [58].

A relatively greater amount of TBW and a relative increase in ICW will result in an underestimation of FM percentage and an overestimation of FFM in the state of morbid obesity. A different body disposition (especially in those with severe abdominal obesity) will result in an overestimation of body fat percentage. Thus, new equations are needed to validate BIA in morbidly obese patients [59].

### 4.2. Eating Disorders: Anorexia

According to the World Health Organization (WHO), Nutrition and Eating Disorders represent one of the most frequent causes of disability in young people in Western countries and have been included among the priorities related to mental health protection as a growing public health problem in all countries (Italian Ministry of Health, 2013). They can be defined as persistent disorders associated with impaired body perception and weight control to the detriment of physical and psychological health. The disease generally appears between the ages of 15 and 19, although in recent times there has been a lowering of the age due to continuous exposure of children, especially through the Web, to “adulting” and aesthetically impossible models. Lack of awareness and an attitude of denial of the problem can slow down referral to treatment. People generally seek treatment at the insistence of a family member or in a situation of serious urgency.

Symptoms to pay special attention to, potentially life-threatening due to severe nutritional impairment, are:

- Significant weight reduction
- Inability to gain body weight
- State of fasting or semi-fasting
- Associated elimination conducts (e.g., vomiting).

Speaking specifically of anorexia nervosa (AN), the key element is severe malnutrition. The low, sometimes absent, awareness of illness results in a low grade of diagnosis and medical treatments. Thus, a lot of attention is paid in the early stages of therapy to increase the patient’s motivation and willingness to change.

**Figure 6:**
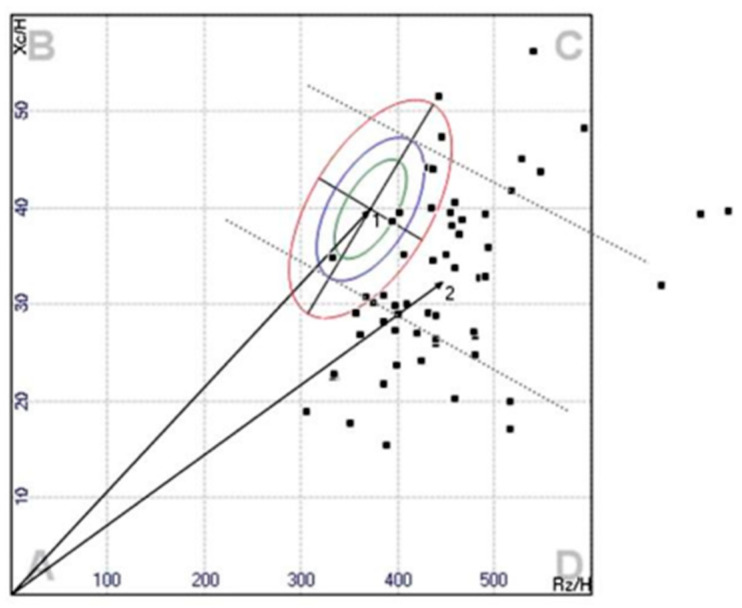
Analysis of bioimpedance vector in patients with anorexia nervosa (AN) Reprinted with permission from Haas et al. [62]. © 2012, John Wiley & Sons, Ltd and Eating Disorders Association.

Bioimpedance testing in this perspective stands both as a tool for assessing the nutritional status of the subject and also as a very strong motivational tool. Bioimpedance measurement is most useful in individuals with Nutrition and Eating Disorders not only for the purpose of assessing their nutritional status in the various stages of re-nourishment, but also to distract them from the strict control of the weight factor, which is no longer seen in absolute terms, but as the result of several compartments, which in turn are indicative of different states of nutrition and health. From this point of view, BIA has also a psychological influence on states of eating disorders and anorexia nervosa, as it can break the obsession with the number displayed on the scale. Through the practice of the impedance technique in a person with Nutrition and Eating Disorder, the human body is perceived as a much more complex machine than the utopian trivialization of weight. Basal metabolism [60], FFM, FM, intra- and extra-cellular fluids, and Phase angle are all terms that gradually enter patients’ daily clinical routine, increasing their interests. Therefore, a critical reading of the performance of the impedance test together with the patient is particularly important and has a terapheutical value [61]. Another study was conducted on 57 women with anorexia nervosa who underwent BIA and BIVA. Twenty-seven women were observed during short-term weight gain (three weeks) and 13 during long-term weight gain (three months) [62]. The purpose of the study was to describe the changes in body composition before and during the re-feeding of anorexic women using both conventional BIA and BIVA, assuming that BIVA would produce more reasonable data than BIA. BIA produced implausible results in 47% of patients. BIVA demonstrated low body cell mass and highly variable ECW volume, ranging from volume contraction to volume expansion at admission and during treatment. BIVA suggested that short-term weight gain consisted mainly of ECW volume, while long-term weight gain involved growth in hydrated cell mass. The results of the study clearly show that BIA in combination with general prediction equations is not a suitable technique for assessing body composition in patients with anorexia nervosa while BIVA offers a good management perspective. This method suggests that short-term re-nourishment consists mainly of increasing extracellular water volume, while tissue restoration occurs with long-term weight gain. Further validation is needed, however, before routinely applying BIVA to monitor qualitative changes in body composition in patients with AN (Figure 6).

It is of interest to evaluate whether the phase angle differs according to the type of underweight in adolescents and young women. For this purpose, skin-flap thickness and BIA (whole body and limbs) were evaluated in three groups of underweight patients (30 with anorexia nervosa, 10 constitutionally thin individuals, and 15 classical dancers) and compared with 30 normal-weight subjects. There were no differences between the three underweight groups with respect to anthropometric and BIA variables with the exception of Phase angle. The latter was significantly higher in dancers, lower in anorexic patients, and no different in constitutionally lean patients. Phase angle, therefore, derived from single-frequency BIA, appears to distinguish between different forms of underweight, confirming itself as an effective indicator of qualitative changes in body composition [63].

### 4.3. Type 1 and 2 Diabetes

Diabetes is a serious condition that can cause catabolism due to loss of muscle protein. It is a chronic disease characterized by an increased concentration of glucose in the blood. This high blood glucose is a sign that the body is unable to maintain the sugar within normal limits, possibly due to a defect in the insulin function or a deficiency of insulin production. Untreated high blood glucose leads over time to chronic complications such as damage to the kidneys, retina, peripheral nerves, and cardiovascular system (heart and arteries).

Diabetics suffer from a frequent state of hyperglycemia, which causes alterations in the distribution of water in the body [64]. In order to assess, through impedance analysis, the relationship between body composition and diabetic disease and to understand possible alterations in water distribution, a study was conducted [65] on 52 patients (eight males, 44 females) with an average age of 46 1/2 years. Of these 15 diabetics, seven had type 1 diabetes, and eight had type 2 diabetes. All patients underwent impedance testing. It was found that diabetic subjects possess less ECW and exchangeable potassium (Ke) in the body than non-diabetics. The causes of this could be altered plasma osmolarity and possible reduction in the mass of metabolically active cells [65].

Evaluation of Phase angle is relevant not only to indicate catabolism in people with diabetes but also to assess differences between different types of therapy. A cross-sectional study was conducted on 182 people with type 2 diabetes and 107 control subjects matched for age and BMI. Phase angle was measured at 5.50 and 100 kHz using multifrequency BIA. Phase angles were compared among different diabetes therapy groups (patients with untreated diabetes, patients receiving oral antidiabetic drugs, and patients receiving insulin therapy). The Phase angle at 100 kHz was found to be a promising measure for assessing catabolic status in people with diabetes [66]. The increased protein breakdown brought about by the condition of insulin resistance induces a decrease in muscle mass in diabetic subjects. BIA is a valid method for monitoring muscle mass. A Japanese study [67] on type 2 diabetics was performed to assess the body composition characteristics of patients already with insulin resistance and cardiovascular risk factors. Comparison with healthy people showed that diabetics were more prone to a decrease in muscle mass and an exercise program was prescribed to prevent muscle degeneration. An additional study [68] showed that diabetics who exercised 30 min a day three times a week were shown to have better glycemic control due to increased muscle mass. In fact, increased muscle mass optimizes insulin release and utilization, consequently lowering blood glucose.

### 4.4. Inflammation

The inflammatory response involves mutations in blood flow, increased vessel permeability, and migration of fluids, proteins, and white blood cells to the site of tissue damage and catabolism [69].

Diet quality, controlling inflammatory biomarkers, is a basic factor in disorders such as obesity, anorexia, and diabetes. One study [70] investigated with bioelectrical impedance parameters the association between dietary inflammatory index (DII) and health characteristics in overweight/obese women. This was a cross-sectional study of 301 participating subjects in which DII was calculated from the food frequency questionnaire (FFQ); body composition was assessed by multifrequency BIA. Depression, anxiety, and stress scale-21 (DASS-21) was the questionnaire used to assess the neurological condition of the participants. A high inflammatory index was calculated in 49% of the participants. Linear regression analysis revealed that FFM and TBW were significantly associated with DII. Thus, it was hypothesized that an anti-inflammatory diet is associated with high FFM and TBW. Indeed, a lower DII value, resulting in less inflammation caused by the diet, could lead to higher FFM and TBW [70]. C-reactive protein (CRP) is a biomarker of meta-inflammation. Since both Phase angle and CRP are influenced by age, BMI, nutritional status, and sex, the following were examined: the association between Phase angle and CRP levels in 1855 subjects (680 males and 1175 females), aged 18 to 59 years, with body mass index (BMI) between 19.5 and 69.4 kg/m^2^. Phase angle values and CRP levels were significantly lower in females than in males (*p* < 0.001), while adherence to the Mediterranean diet (MD) was lower in males than in females (*p* < 0.001). After adjustment for age, physical activity, body mass index, waist circumference, and MD adherence, Phase angle remained negatively associated with CRP levels in both sexes (*p*< 0.001). In ROC (Receiver Operating Characteristic) analysis, Phase angle ≤ 5.5° in males and ≤5.4° in females were found to be threshold values of increased high sensitivity (hs)-CRP levels [71]. The study thus suggested that the Phase angle may be a valid predictor of CRP levels in both sexes regardless of body weight and MD adherence, avoiding both blood sample collection and expensive biochemical testing.

### 4.5. Cancer

BIVA analysis allows for a detailed estimation of the patient’s hydration status and cell mass. As mentioned, the axis identifying the body’s cell mass flows from the upper left quadrant to the lower right quadrant indicating a change in X_c_ and an increase or decrease in cell mass. The three tolerance ellipsoids allow the measurement data of an individual to be compared with the reference range of the normal population, which are in the range of the 50% ellipse. Strong outliers are visible in the 95% ellipse and outside of it. If the values are at the end of the vector in the lower quadrant of the reference range, we are in the presence of hyperhydration; at the end of the vector in the upper quadrant, they indicate strong dehydration. Cell loss, which can occur during malnutrition, can be seen with values within the lower right quadrant; values in the upper left quadrant indicate an increase in cell mass.

BIVA and Phase angle can thus provide essential information on the body composition of cancer patients. Accurate estimation of the nutritional status of such patients plays a vital role in prognosis and further therapeutic procedures. Cancer patients may suffer from so-called cancer cachexia, which is a multifactorial syndrome distinguished by unwanted weight loss, loss of muscle mass, fatigue and weakness, and visibly reduced appetite.

The following were evaluated: seven male and three female populations with cancer pathology. The majority (*n* = 5) presented normal body composition, followed by cachexia (*n* = 4) and athletic (*n* = 1). The change in body composition appeared to be related to gender, disease type, and severity. The BIVA RX_c_ z-score method proved to be valid for studying body composition, according to cancer type, stage, sex, and ethnicity. The limitations of the method mainly relate to variability among bioimpedance analyzers. Better assessment of body composition has the potential to personalize therapeutic, nutritional, and hydration management [72]. Tracking the trajectory of vector displacement toward the position of the target reference vector can provide useful feedback in planning the supportive therapy of individual patients [73].

Relative to colorectal cancer, 52 patients, in histologically confirmed stage IV, were evaluated through BIA and Phase angle. Patients with a Phase angle < or = 5.57 had a median survival of 8.6 months, while those with a Phase angle >5.57 had a median survival of 40.4 months. Thus, Phase angle is a valid prognostic indicator in patients with advanced colorectal cancer [74].

A study conducted on 58 patients undergoing three-week inpatient rehabilitation after acute prostate cancer therapy included BIVA and hand grip strength test (HGS) at the beginning and end of treatment. The BIVA model and the prevalence of critically low Phase angle values highlight the need for intensive rehabilitation and stand as a valuable aid in personalizing and optimizing the treatment [75].

### 4.6. Diuretic Therapy

Diuretics can be used in a variety of clinical conditions, including edema, ascites, and diseases related to the kidney, liver, and heart. Edema indicates abnormal retention of sodium and water. Heart failure is characterized by significant body fluid retention leading to anasarca and acute pulmonary edema. The use of BIVA for the assessment of hydration status plays an important role in the prognosis of acute heart failure, in the diagnosis of dyspnea, and also in nephropathy caused by contrast medium following invasive procedures such as coronary angiography and angioplasty, and to define diuretic therapy. In the past decade, cardiology has increasingly relied on innovative methods for the diagnosis of cardiovascular diseases, including the assessment of soft tissue hydration status by noninvasive total body vector analysis with a BIVA instrument.

A total of 487 patients with acute decompensated heart failure (ADHF) and 413 with chronic heart failure underwent BIVA testing and the result was compared with brain natriuretic peptide (BNP) testing. BIVA was more accurate than BNP in detecting peripheral congestion in both patients with ADHF (AUC 0.88 vs. 0.57 respectively *p* < 0.001) and with chronic decompensation [76]. The determination of dry weight in patients with terminal kidney disease undergoing extracorporeal dialysis treatment has always been a subject of research [77].

BIVA was validated in 1994 by an Italian study published by Kidney International [26]. Patients on extracorporeal dialysis were recruited. Specifically, 217 adult subjects were divided into four groups: 86 healthy patients (38 males, 48 females, age range 16–66 years), 55 patients (31 males, 24 females, age range 18–75 years) with mild to terminal chronic renal failure (CRI) under conservative treatment with undetectable to severe edema (15% with apparent edema), 36 patients (19 males, 17 females age 16 to 75 years) with idiopathic nephrotic syndrome (NS) with undetectable to severe edema (58% with apparent edema), and 40 obese subjects (nine males, 31 females, age 24 to 71 years) with BMI >31 kg/m^2^, free of diabetes, renal, cardiac, and hepatic diseases. Bioimpedance testing was performed before and after the dialysis sessions, and the bioelectrical data (of R and X_c_ obtained were compared with the values of the healthy population [26].

In conclusion, the study highlighted a bioimpedance threshold for fluid overload and validated a useful novel graphical method for the identification, monitoring, and therapy planning of renal patients with altered fluid balance, using direct bioimpedance measurements and following a novel bivariate vector approach. While highlighting the need for further rigorous studies to define whether the method proves to be generally applicable to a variety of different clinical situations, the study demonstrates the potential of the method in those patients in whom basal impedance vectors have already been measured under healthy conditions, so that subsequent changes from their own smaller tolerance ellipses could be significant even if they remained within the 75% tolerance ellipse of the reference population.

### 4.7. Bariatric Surgery

**Figure 7:**
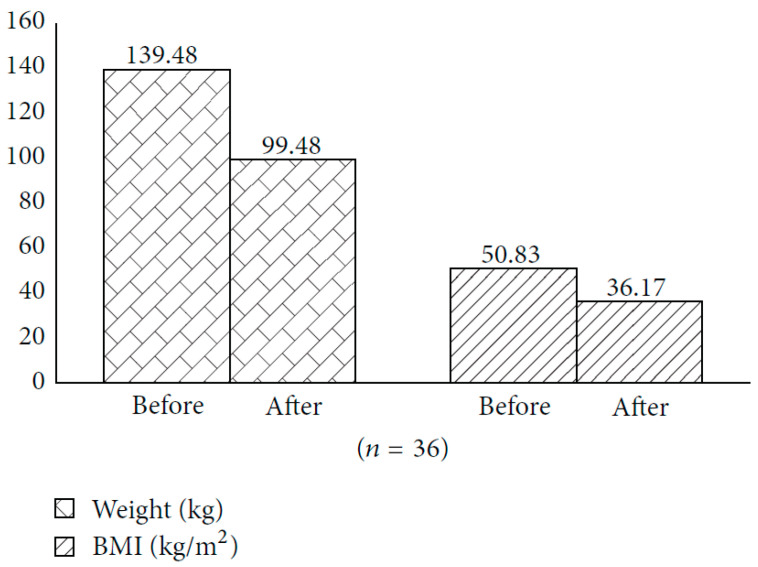
Weight and BMI before and six months after bariatric surgery. Reprinted from Wilson Rodrigues de Freitas Junior et al. [78]. © 2014, Wilson Rodrigues de Freitas Junior et al.

Morbid obesity can cause up to a 22% reduction in life expectancy. Bariatric surgery is a type of surgery used to treat level II and III obesity in those patients in whom conventional clinical treatments are unsuccessful. Following gastric bypass surgery, the patient in fact achieves substantial weight loss within the first year of treatment. In order to assess the quality of weight loss recorded in the first six months following bariatric surgery, 36 patients underwent BIA to assess FM and FFM before and after surgery. An average weight loss of 28.6 percent (40 kg) was found six months after surgery (Figure 7). Along with the reduction in total body weight, however, analysis of different body weight components by bioelectrical impedance testing also revealed an unwanted loss of FFM [78].

Standard methods for body composition assessment prove ineffective for analyzing the quality of weight loss in individuals with severe obesity. Particularly in grade III obese patients, standard body composition assessment methods prove to be unreliable for analyzing the quality of weight loss after bariatric surgery. In 43 obese grade III women undergoing bariatric surgery, BIVA and RX_c_ plot were then used [79]. Similar to the previously mentioned study, the patients’ anthropometric and bioimpedance data were obtained before the operation and reevaluated long-term over the following four years. Placing the vectors in the cachexia and water retention quadrant (lower right quadrant of the graph) throughout the postoperative period showed that changes in body composition and weight loss affected both FM and FFM. Therefore, it is necessary that protein supplementation and physical activity are performed following bariatric surgery in order to minimize muscle mass loss [79].

### 4.8. Therapeutic Fasting

Therapeutic fasting is a radical intervention that, under careful medical supervision, can be used as an alternative to bariatric surgery on severely obese subjects, or even to support the surgical preparatory phase. A study of six obese women undergoing a two-week therapeutic fast involved daily bioelectrical impedance measurements and TBW assessment before and after the end of the fast. Fasting was followed by a phase of progressive re-feeding (1200-calorie glucose solution on the first day, low-fat diet on subsequent days). R, X_c_, and impedance (Z) as a function of the square of height were assessed each morning as suggested by Hoffer et al. [6] and Lukaski et al. [9]. Data from the first week of fasting were compared with those from the second week by evaluating changes in TBW. During the first week, their weight loss (10 kg on average) was observed, followed by stabilization in the second week. R, X_c_, and impedance also increased particularly during the first week. A decrease in R and X_c_ during re-feeding and a decrease in TBW and h^2^/R during the entire fasting period was observed. This occurred in all patients except the one with more severe obesity. This leads one to believe that BIA returns satisfactory results, particularly in moderately obese subjects.

The rapid increase in R, X_c_, and Z during the first week and their relative stability during the second week confirm that the most consistent change in TBW occurred during the first seven days of fasting. However, the study showed that if body water changes disproportionately to FFM, the BIA should be interpreted with caution as the measurements would not accurately reflect changes in FFM [80].

## 5. Conclusions

Several studies have demonstrated the prognostic impact of the Phase angle in different clinical settings. BIVA has been shown to provide information on hydration and body cell mass, thus enabling the evaluation of patients in whom body composition calculation fails due to impaired hydration.

In particular, in physiological conditions, the analysis of classical and specific BIVA:

- has been found to be remarkably effective in competitive sports, in assessing maturity differences in the body composition of young athletes, and in obtaining an athlete-specific profile [35,37];
- to date represents the quintessential technique for evaluation in pregnancy as it allows monitoring of hemodynamic adaptation and identification of patients at risk for the onset of hypertensive pathology [41];
- confirms its validity in menopause, in relation to the strong changes in the hydro electrolyte picture both for evaluation of fat tissue redistribution and for predisposition of hormone replacement therapy [48];
- stands as an indispensable investigative and diagnostic aid for the detection of weight and hydration changes in elderly subjects, as it is valid even without the active cooperation of the patient and in support of evaluative protocols [49,50,55].

Under pathological conditions, with regard to classical and specific BIVA, it was demonstrated that:

- Phase angle assessment is relevant in diabetic patients with type 1 and type 2 diabetes to detect catabolism but also to assess differences between different types of therapy [65,66,67,68];
- Phase angle is a valid predictor of CRP levels, a biomarker of meta-inflammation. Thus, bioelectrical impedance parameters are extremely useful in the detection of inflammatory indices [69];
- BIVA and Phase angle can provide essential information on the body composition of cancer patients, particularly those with cachexia. The validity of the method extends from prognosis to therapeutic procedures [71,72,73,74];
- BIVA is useful in diseases requiring diuretic therapy, for the detection of fluid overload and the identification, monitoring, and therapy planning of renal patients with impaired fluid balance. However, the greater potential of the method has been found in those patients in whom basal impedance vectors had already been measured under healthy conditions [26,75];
- In patients with average and mild obesity, BIVA was proved to be a valid method of analysis, both prognostic and therapeutic. Nonetheless, significant limitations of BIVA method in morbid obesity type III have been detected as the underestimation of body fat percentage and overestimation of FFMv [58,59,60,79]; in extreme interventions for the treatment of obesity, such as bariatric surgery, BIVA was shown to be useful in highlighting a loss of FM, as well as of FFM [78]; on the other hand, following therapeutic fasting, the aforementioned limitations were confirmed in the evaluation of subjects with severe obesity [79];
- In the assessment of the body composition of subjects with anorexia nervosa, BIVA assumes considerable importance at the psychological level, while the results show significant limitations for BIA [62,63].

## Data Availability

Not applicable.
